# The changing malaria trend and control efforts in Oromia Special zone, Amhara Regional State, North-East Ethiopia

**DOI:** 10.1186/s12936-022-04149-y

**Published:** 2022-04-22

**Authors:** Selomon Tefera, Temesgen Bekele, Kefelegn Getahun, Abiyot Negash, Tsige Ketema

**Affiliations:** 1 College of Natural Sciences, Department of Biology, Jimma University, Jimma, Ethiopia; 2 College of Social Sciences and Humanity, Department of Geography and Environmental Studies, Jimma University, Jimma, Ethiopia; 3 College of Natural Sciences, Department of Statistics, Jimma University, Jimma, Ethiopia

**Keywords:** IRS, LLIN, Malaria, Oromia Special zone, *Plasmodium falciparum*, *Plasmodium vivax*, Prevalence

## Abstract

**Background:**

Countries in malaria endemic regions are determinedly making an effort to achieve the global malaria elimination goals. In Ethiopia, too, all concerned bodies have given attention to this mission as one of their priority areas so that malaria would be eradicated from the country. Despite the success stories from some areas in the country, however, malaria is still a major public health concern in most parts of Ethiopia. Therefore, this study is aimed at analysing the changing malaria trend and assessing the impact of malaria control efforts in one of the malaria endemic regions of Ethiopia.

**Methods:**

Five years data on clinical malaria cases diagnosed and treated at all health facilities (including 28 Health Centres, 105 Health Posts and 2 Hospitals) in Oromia Special zone, Amhara Regional State, Ethiopia, were reviewed for the period from June 2014 to June 2019. Data on different interventional activities undertaken in the zone during the specified period were obtained from the Regional Health Bureau.

**Results:**

The cumulative malaria positivity rate documented in the zone was 12.5% (n = 65,463/524,722). *Plasmodium falciparum* infection was the dominant malaria aetiology and accounted for 78.9% (n = 51,679). The age group with the highest malaria burden was found to be those aged above 15 years (54.14%, n = 35,443/65,463). The malaria trend showed a sharp decreasing pattern from 19.33% (in 2015) to 5.65% (in 2018), although insignificant increment was recorded in 2019 (8.53%). Distribution of long-lasting insecticidal nets** (**LLIN**)** and indoor residual spraying (IRS) were undertaken in the zone once a year only for two years, specifically in 2014 and 2017. In 2014, a single LLIN was distributed per head of households, which was not sufficient for a family size of more than one family member. Number of houses sprayed with indoor residual spray in 2014 and 2017 were 33,314 and 32,184 houses, respectively, leading to the assumption that, 151,444 (25.9%) and 141,641 (24.2%) population were protected in year 2014 and 2017, respectively. The analysis has shown that *P. falciparum* positivity rate was significantly decreased following the interventional activities by 3.3% (p = 0.009), but interventional efforts did not appear to have significant effect on vivax malaria, as positivity rate of this parasite increased by 1.49% (p = 0.0218).

**Conclusion:**

Malaria burden has shown a decreasing pattern in the study area, although the pattern was not consistent throughout all the years and across the districts in the study area. Therefore, unremitting surveillance along implementation of interventional efforts should be considered taking into account the unique features of *Plasmodium* species, population dynamics in the zone, seasonality, and malaria history at different districts of the zone should be in place to achieve the envisaged national malaria elimination goal by 2030.

## Background

Ethiopia is one of the rare African countries where the two principal human malaria parasites, *Plasmodium falciparum* and *Plasmodium vivax* co-exist [1]. According to the National Malaria Strategic Plan, and a nationwide systematic review and meta-analysis, nearly 31% of malaria cases in Ethiopia were due to *P*. *vivax*, while 69% were due to *P*. *falciparum* [2, 3]. Majority of the country’s land coverage were malarious and about 60 million people are estimated to be at risk of malaria [2], although the burden has been decreased in most endemic areas [4].

The country has a firm stand to achieve the malaria eradication goal set by World Health Organization by 2030 [5]. In line with this, it had clearly declared a malaria elimination goal from 239 chosen low transmission districts located in six different regions of the country [6]. To achieve this ambitious goal, the public health authorities in Ethiopia have been engaged in the enforcement of massive interventional activities [4]. Some of the tools employed in the intervening activities were: the use of insecticide-treated bed nets (ITNs), indoor residual spraying (IRS), rapid diagnosis and treatment with artemisinin-based combination therapy, periodic inspection and continuous nation-wide malaria surveillance, enhancing awareness of treatment seeking behaviour of the population. Furthermore, malaria situation analysis programme have been in practice for long at most malaria endemic regions of the country [4, 5].

Some of the considerable successes obtained at national level so far were enhancement of malaria confirmatory test from 53% (in 2012) to 88.3% (in 2018) [1]. In years between 2015 and 2018, Ethiopia has attained about 57% decline in malaria incidence and 54% reduction in malaria associated deaths [7] This was further supported by evidences from a study that reported drops in malaria morbidity and mortality by more than 88% and 96.5%, respectively, in the last 25 years [8].

In most of the national malaria endemic regions, reports show that malaria burden has shown a declining tendency [4, 8, 9]. However, this reduction is uneven throughout the country as different regions have been responding differently. In some parts, the malaria prevalence dropped to < 1% [9], while in others, the number of malaria cases being recorded remain substantially high [10, 11]. For the same reasons, in some regions/districts malaria is still a major public health concern calling for an immediate action by concerned public health authorities.

Oromia Special Zone in Amhara Regional State of Ethiopia is one of the regions known for its malaria endemicity in the country. Although the magnitude of malaria cases varies across the different districts in the zone, there is no comprehensive report on the status of malaria prevalence and interventional activities undertaken in the zone to control malaria related deaths. Therefore, this study was aimed at analysing the changing malaria trend and assessing the real impacts of malaria intervention strategies in the special zone.

## Methods

### Description of the study area

**Fig. 1:**
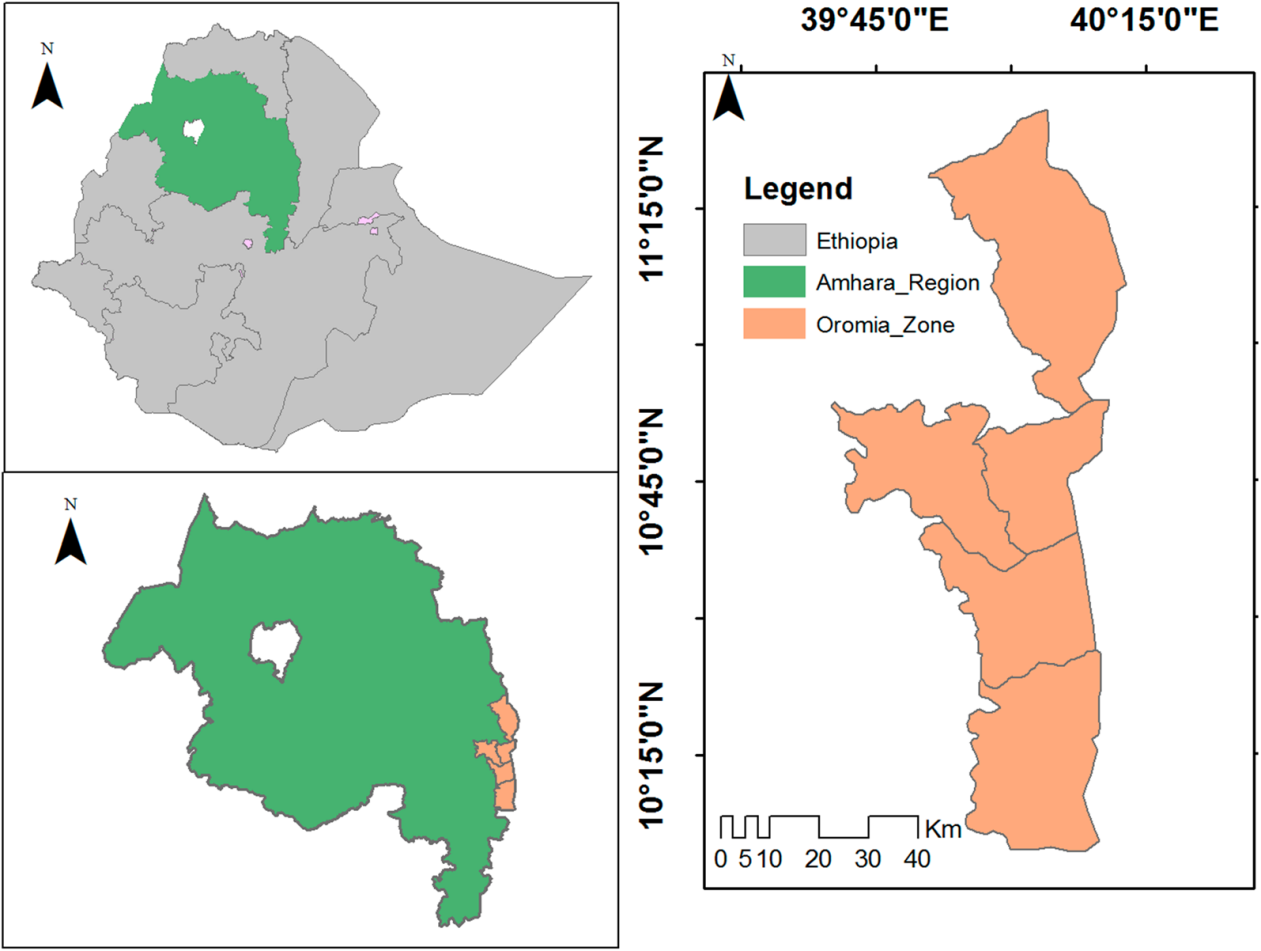
Study sites

The study was conducted in Oromia Special Zone of Amhara Regional State, located in northern part of Ethiopia. The zone has a total population of 594, 265 [12]. Geographically, the study site is located at latitude and longitude of 10°45'N 39°45'E, and 11.15′0°N 40.15′0°E, respectively, and within an altitude of 1424 m. The zone has accommodated all climatic zones (arid, semi-arid semi-humid, and humid). The zone has seven Woredas/Districts namely Artuma-Fursi, Bati, Dewachefa, Dewe- Harewa, Jilie-Timuga, Kemissie and Bati Towns. All health facilities (28 health centres, 105 health posts and 2 hospitals) found in the zone were included in the study and data was collected from all these health facilities (Fig. 1).

### Study design and data collection procedure

A facility based retrospective study design was employed. Medical records of all patients diagnosed and received treatment for malaria at all health facilities of the zone between the years 2014 and 2019 were reviewed. Data on socio-demographic characteristics, season at which the patients’ visited health facilities, and the types of malaria parasite (*Plasmodium* spp.) responsible for the infections were collected on a pre-designed data collection tool. In addition, information on the interventional activities executed in the zone between 2014 and 2019 were collected from the Zonal Health Bureau.

### Data analysis

All data retrieved from the medical records were entered into Microsoft excel sheet (Window 2016) and checked for completeness. Then, data were exported to R-software (version 4.0.0). Descriptive statistics was used to analyse data on socio-demographic characteristics of malaria patients, seasonality of malaria infection rate and *Plasmodium* species responsible for the observed infections or cases. Slide positivity for each year was computed by dividing number of malaria positive cases to the total population examined [+ ve/total examined]. Multiple linear regression model was used to analyse impact of interventional activities on malaria trend from multiple input variables. Interrupted time series analysis (ITS) was used to track malaria infection rate trend for a period before and after a point of intervention (IRS and LLIN distribution), and its impacts on malaria trend. Hence, standard ITS analyses segmented regression model used was:

$$ Y_{t} = \beta_{0} + \beta_{1} year + \beta_{2} Months + \beta_{3} P.fal + \beta_{5} Underfive + \beta_{6} five\,to\,fourteen + \beta_{7} more\,than\,fourteen + \beta_{8} Interv. $$

## Results

### Malaria prevalence

In the study years, 2014–2019, a total of 524,722 clinically suspected malaria cases were diagnosed using a microscopy (80.07%) and RDT (19.92%) in the health facilities. Among the suspected cases, 65,463 (12.5%) were found positive for malaria and 99.8% of them were out-patients. Based on the current analysis, the overall malaria positivity rate over the 6 years was 0.115, and the highest positivity (0.14 to 0.18) was noted during 2014 to 2016 period. In the recent years, the positivity rate has shown a declining pattern. Infection refereeing to *Plasmodium* species had shown that 51,679 (79%) were due to *P. falciparum* while 21% (n = 13,657) were due to *P. vivax* (Table 1). Malaria prevalence among pregnant women in across the six years period was 3.65% (n = 503).

**Table 1:** Prevalence of malaria between 2014 and 2019 in Oromia Special Zone, Amhara Regional State, North Ethiopia

Year	Parasitologically examined	Confirmed	Slide positivity rate	*P. falciparum*	*P. vivax*	Mixed infection*
2014	82,986	13,795 (16.6)	0.16	10,667 (77.33)	3086 (22.37)	42 (0.30)
2015	101,531	19,627 (19.33)	0.18	15,878 (80.9)	3702 (18.86)	47 (0.24)
2016	95,559	13,898 (14.55)	0.14	10,332 (74.3)	3566 (25.65)	4 (0.03)
2017	78,716	6017 (7.64)	0.08	4257 (70.75)	1758 (29.22)	2 (0.033)
2018	69,987	3937 (5.63)	0.05	3193 (81.1)	738 (18.75)	6 (0.15)
2019	95,943	8185 (8.53)	0.08	7352 (89.8)	807 (995)	26 (0.32)
Total	524,722	65,463 (12.5)	0.115	51,679 (79)	13,657 (21)	127 (0.2)

^*^mixed infection refers to infection with both *P. falciparum* and *P. vivax*

**Fig. 2:**
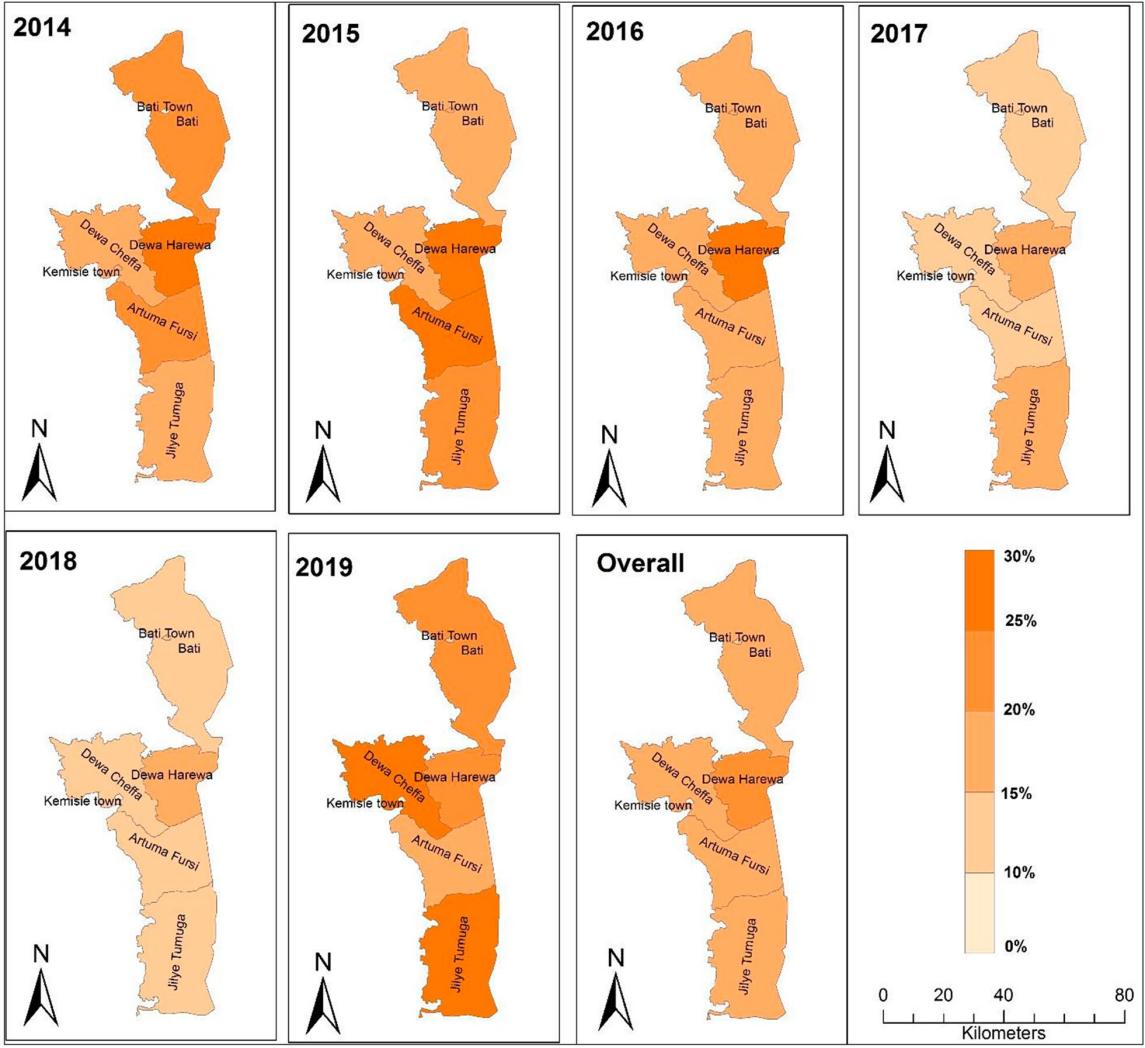
Malaria prevalence at districts between 2014 and 2019 in Oromia Special zone at Amhara regional state, Ethiopia

Overall, malaria burden has shown variability from district to district, and from year to year. Relatively the highest malaria prevalence, 28.56%, 29.08% and 27.1%, was recorded in Dewa Harewa in years 2014, 2015 and 2016, respectively. The current comprehensive study done for the period that lasts from 2014 to 2019 also demonstrated the highest malaria burden in the same district (22.62%), followed by districts named Jilie Timuga and Artuma Fursi (16% each). Comparatively, Bati and Kemissie towns had the lowest malaria burden in these years (Table 2). No death cases were reported from any one of the districts during the 2014 to 2019 period. In the following illustration, a steadily declining malaria prevalence was noted between 2014 (20–30%) and 2018 (< 10%) in all the districts, except in Daewa Harewa, where more than 10% malaria prevalence (10.26%) was recorded. In the most recent year, 2019, for some reasons, the epidemiology of malaria in all districts of the zone has shown a slight increment (10–20%), though insignificant (Fig. 2).

**Table 2:** Malaria prevalence at different districts of Oromia Special zone, Amhara Regional State between 2014 and 2019, North Ethiopia

Districts	Year													
	2014		2015		2016		2017		2018		2019		Overall	
	Test	+ ve (%)	Test	+ ve (%)	Test	+ ve (%)	Test	+ ve (%)	Test	+ ve (%)	Test	+ ve (%)	Total	+ ve
													test	(%)
Artuma Fursi	17,615	3751 (21.29)	19,159	5237 (27.33)	13,466	2024 (15.03)	8829	595 (6.74)	7577	235 (3.1)	12,394	764 (6.16)	79,040	12,606 (16)
Bati Town	9096	862 (9.48)	15,042	2195 (14.59)	15,663	1921 (12.26)	16,865	1000 (5.93)	13,298	688 (5.17)	18,767	651 (3.67)	88,731	7317 (8.24)
Bati Wereda	5904	1358 (23)	7268	1421 (19.55)	6857	1102 (16.07)	6382	588 (9.21)	6,731	438 (6.51)	8887	877 (9.87)	42,029	5784 (13.76)
Dewachefa	11,683	1437 (12.3)	10,193	1773 (17.39)	11,008	2166 (19.7)	9597	553 (5.76)	7,193	282 (3.92)	11,590	1,604 (14.1)	61,264	7815 (12.76)
Dewa Harewa	2955	844 (28.56)	4494	1307 (29.08)	3424	929 (27.1)	1703	294 (17)	1,833	188 (10.26)	2262	209 (9.39)	16,671	3771 (22.62)*
Jilie Timuga	20,529	3942 (19.2)	26,751	5801 (21.7)	22,794	3567 (15.64)	17,735	2179 (12.3)	18,066	1660 (9.19)	24,197	3540 (14.63)	130,072	20,689 (16)
Kemissie Town	15,204	1601 (10.5)	18,424	1893 (10.27)	22,551	2193 (9.72)	17,605	808 (4.59)	15,289	446 (2.92)	17,846	540 (3.21)	106,919	7481 (7)
Total	82,986	13,837 (16.7)	101, 531	19,580 (19.94)	97,559	13,898 (16.5)	78,716	6017 (8.82)	69,987	3937 (5.86)	95,943	8185 (8.53)	524,722	65,463 (12.5)

^*^ = indicates a district with highest overall prevalence

**Fig. 3:**
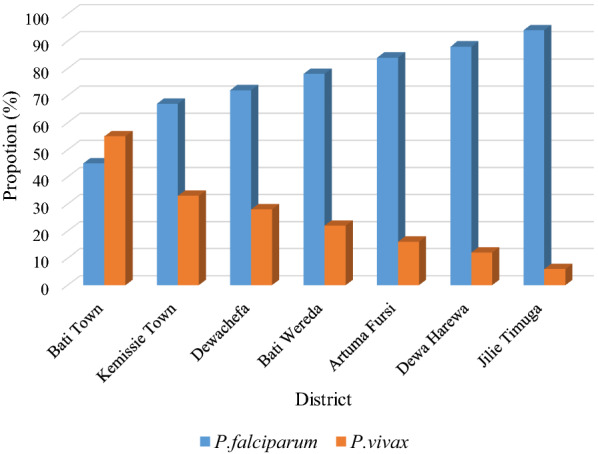
Proportion of *P. falciparum* and *P. vivax* infections between 2014 and 2019 at different districts of Oromia special zone in Amhara regional state, Ethiopia

The composition of prevalent *Plasmodium* species varied from district to district. In Jilie Timuga and Dewa Harewa, *P. falciparum* was the dominant malaria parasite, which was estimated to cause 93.8% and 87.8% of malaria in these two districts, respectively. Whereas, in Bati town the two *Plasmodium* species (both *P. falciparum* and *P. vivax*) were equally prevalent, where *P. falciparum* accounted for 45% of malaria cases while 55% malaria cases were accounted to *P. vivax*. Except for the slight differences noted in Kemissie Town, the share of the two *Plasmodium* species were similar in the remaining districts. Overall, *P. falciparum* was the dominant malaria parasite in the zone (Fig. 3).

Analysis of the status of malaria among patients of different age groups revealed the highest positivity rate of 54.14% [ranged from 51.4% (2019) to 55.3% (2015)] in adult patients (≥ 15 years). Whereas, malaria share among younger children, aged < 5 years, who were supposed to be biologically at risk to malaria, was comparatively lower (17.38%) than other age groups (Table 3).

**Table 3:** The status of malaria among different age groups between 2014 and 2019, Oromia Special Zone, Amhara Region, North Ethiopia

Patients category (year)	Year						Total No. (%)
	2014 No. (%)	2015 No. (%)	2016 No. (%)	2017 No. (%)	2018 No. (%)	2019 No. (%)	
< 5	2272 (16.5)	3121 (15.9)	2414 (17.4)	1124 (18.7)	774 (19.7)	1677 (20.5)	11,382 (17.38)
5–14	3906 (28.1)	5653 (28.8)	4050 (29.1)	1683 (28)	1046 (26.6)	2300 (28.1)	18,638 (28.47)
≥ 15	7617 (55.21)	10,871 (55.3)	7438 (53.5)	3210 (53.3)	2117 (53.8)	4208 (51.4)	35,443 (54.14)
Total	13,795	19,627	13,902	6,017	3937	8,185	65,463

**Fig. 4:**
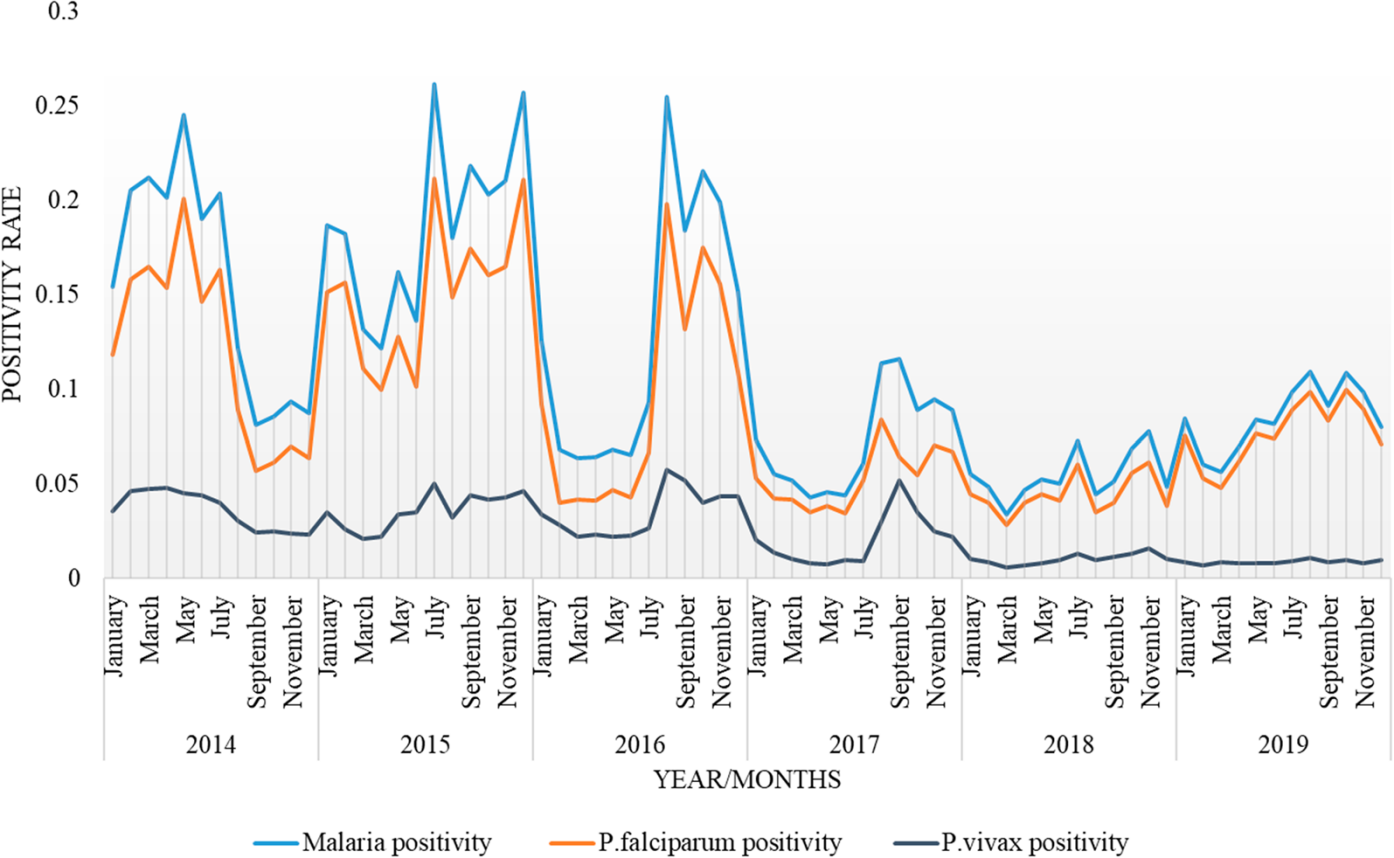
Monthly malaria pattern between 2014 and 2019 at Oromia Special zone, Amhara regional state, Ethiopia

Malaria positivity rate had shown a diminishing trend between 2014 and 2019 with unpredictable peak seasons in different years. In 2014, malaria prevalence reached its peak during March to May, whereas in the years, 2016 to 2019, the highest malaria infection was documented in months from June/July to September/October, known to be the rainy season in the special zone. In the year 2015, however, irregular peaks were observed at different months of the year. The prevalence of malaria was proportionately lower during dry season (January to March) in all years considered in this study. The mean malaria cases recorded from June to October (12.7 to 13.54%) for all individual years were higher than the overall malaria prevalence (12.5%) documented (Fig. 4).

### Malaria control efforts

As per the information obtained from the Zonal Health Bureau, IRS and LLIN were distributed once per year for only 2 years, specifically in 2014 and 2017. The number of houses sprayed with IRS in 2014 and 2017 were 33,314 and 32,184, respectively. The assumption made during the spraying of houses was protection against malaria of about 151,444 (25.9%) and 141,641 (24.2%) people living in the zone in the year 2014 and 2017, respectively. Similarly, 271,719 LLIN were distributed in 2014 to address 116,839 houses, and protect a round 509,864 population. Likewise, in 2017, a total of 282,451 LLIN were distributed addressing 132,273 houses. The sum of number of LLIN distributed per head of household could cover around 93.25% (554,170/594,265) of population in the zone since 2014 (Table 4).

**Table 4:** IRS and LLIN coverage of different districts between 2014 and 2019 in Oromia Special Zone, Amhara Regional State, North Ethiopia

Year	District	IRS		LLIN	
		Houses sprayed	Population protected	LLIN distributed	Population protected
2014	Artuma Fursi	7377	28,343	50,088	97,443
	Bati Town	1693	8350	18,300	34,480
	Bati Wereda	4373	21,980	42,480	92,944
	Dewachefa	7908	37,005	69,045	114,905
	Dewa Harewa	3349	15,600	27,600	47,941
	Jilie Timuga	5212	20,518	44,257	84,963
	Kemissie Town	3402	19,644	19,949	37,168
	Total	33,314	151,444	271,719	509,864
2015	NI	NI	NI	NI	NI
2016	NI	NI	NI	NI	NI
2017	Artuma Fursi	6933	31,243	53,700	105,565
	Bati Town	2084	10,454	16,677	36,363
	Bati Wereda	4048	22,455	47,114	98,019
	Dewachefa	6310	29,017	70,377	151,645
	Dewa Harewa	3439	13,600	29,353	48,563
	Jilie Timuga	5020	23,235	45,442	93,073
	Kemissie Town	4350	11,637	19,788	42,853
	Total	32,184	141,641	282,451	568,781
2018	NI	NI	NI	NI	NI
2019	NI	NI	NI	NI	NI

*NI* no intervention

The coverage status of IRS and LLIN at different districts of the zone has shown that the highest intervention was carried out at Dewachefa and Artuma Fursi districts, whereas the lowest intervention was given to Bati Town in both years (2014 and 2017). Concerning IRS coverage, Artuma Fursi and Dewachefa had got the highest number of LLIN in both years (2014 and 2017). In these two districts, the presumption is that the number of protected populations with LLIN were also higher than other districts. Relative prevalence of malaria in these districts was high, with 12.65 and 15.83% prevalence at Artuma Fursi and Dewachefa, respectively. However, district with the highest malaria between 2014 and 2019, specifically Dewa Harewa (22.53%) (Table 2) had received low number of LLIN and only few houses in the district were addressed with IRS, compared to other districts. On the contrary, the two towns, Bati and Kemissie had got fewer number of LLIN and the number of population protected in 2014 and 2017 were also minimal (Table 4).

### Impact of control efforts on malaria trend

Effects of seasonal variability, interventional activities and demographic variable (including age) on the trend of malaria infection rate, in general, and *P. falciparum* positivity rate, in particular, has shown a significant declining trend with drop down to prevalence of only 3.3% (p = 0.009) following the interventions. Whereas, *P. vivax* infection rate has showed an incremental trend mainly in the month of September, with increment of 1.49% (p = 0.0218), further confirming a lack of significant interventional impact (0.4%, p = 0.167) on the vivax malaria. On the other hand, although the highest malaria positivity rate was documented among adult patients (age > 14 years), control efforts did not show significant impact on patients of different age groups.

**Fig. 5:**
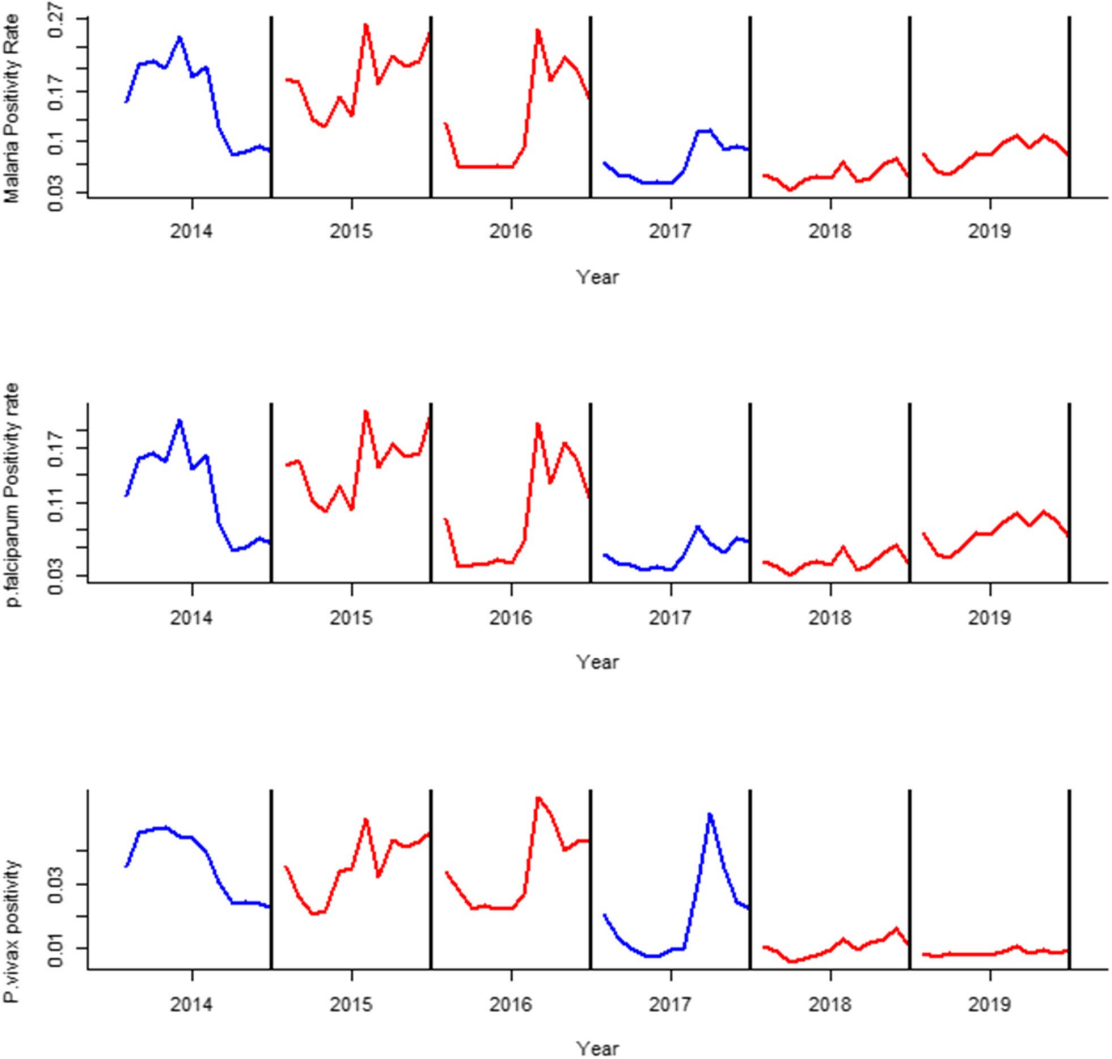
Interrupted time series for malaria, *P. falciparum*, and *P. vivax* positivity rate between 2014 and 2019 in Oromia Special zone at Amhara regional state, Ethiopia

Based on ITS analysis, the control efforts implemented in 2017 had shown an observable reduction in positivity rate for malaria infection in general and *P. falciparum* in particular. In the following two years (2018 and 2019), the reduction pattern was persistent for all cases including *P. vivax*, although considerable reduction did not occur for vivax malaria in the year 2017 (Fig. 5).

## Discussion

This study was carried out in Oromia Special Zone of Amhara Regional State, North Ethiopia, primarily aimed to assess the prevalence of malaria among in-patients and out-patients admitted to all health facilities of the zone and control efforts undertaken in the zone between 2014 and 2019. The study analysed all patients’ recorded data. Consequently, a total of 524,722 clinically suspected malaria cases were diagnosed among samples analysed from all health facilities in specified years. Out of the total cases, 65,463 (12.5%) were found positive for malaria. Finding of the study demonstrated that malaria positivity rate has declined from 19.33% (in 2015) to 5.63% (in 2018) in the study area. In contrast, the ten years reports from Oromia Regional State, particularly Asendabo Health Centre, in Jimma zone, reduction in positivity rate was relatively swift (reduce from 27.9% to 1.1%) [9], but comparable to the 7 years reports (2012–2018) made from the neighbouring Tigray Region (6.96%) [10, 11, 13].

The National Malaria Control and Elimination Programme has set a goal to lower malaria cases by 40% from the baseline by 2020 and eliminate malaria from Ethiopia by 2030 [14]. The malaria positivity rate reduction observed in the study area, might suggests the possibility of achieving this ambitious goal within the anticipated time. This is mainly due to the sustainably wider coverage of interventional efforts being undertaken in most malaria endemic regions of the country. Ethiopia set a strategic plan to eliminate malarial, which was built up on the achievement made so far through immense distribution of LLIN and implementation of IRS, increased diagnosis and case detection, increased access to treatment, and community mobilization effort [15, 16]. In agreement with these national efforts, the zone has distributed LLIN and spraying insecticides (IRS) once a year for two non-consecutive years, mainly in 2014 and 2017 [17], although it was not continued since then. The intervention might be considered as one of the major factors that contributed to the observed reduction in malaria burden documented in the study area. Accordingly, relative risk of malaria infection, mainly *P. falciparum,* had shown strong significant reduction following the implementation of interventional activities. However, the overall malaria burden showed significant variability in magnitude/burden from district to district, and from year to year. In Dewa Harewa, even though the number of reported cases was lower as compared to other districts (Artuma Fursi, Jilie Timuga and Bati Woreda), the overall recorded malaria positivity rate over the period of 2014 to 2019 was the highest (22.62%), followed by the case in Jilie timuga and Artuma fursi (16% each). This might be attributed to the inconsistent and limited number of LLIN distribution events and implementation of IRS in this district, compared to others districts.

Unlike other areas, mainly central part of Ethiopia where *P. vivax* is the dominant malaria parasite [18], in this zone *P. falciparum* was the dominant one in most districts and responsible for the majority of malaria (79%) in the study area. This might be attributed to the hot climatic condition of the area, where 85% of the total zone is low land (locally called Kolla). The average annual temperature and means rainfall of the region were reported to be > 27 °C and about 510 mm, respectively (Ethiopian Climate and Seasons (www.ethiopiantreasures.co.uk). Such climatic conditions could favour the incubation of *P. falciparum* in the vector, more than *P. vivax* [19]. On the other hand, results of the multiple linear regression analysis revealed that *P. vivax* did not show significant response to the control efforts in the zone although the trend seems decreasing. This is mainly because the two malaria control methods, ITN and IRS, are not always as effective against *P. vivax* as they are against *P. falciparum* [20]. Some important vectors of *P. vivax* in malaria endemic areas have primarily early-biting, outdoor-feeding and outdoor-resting behaviours [21]. The ability of vector control to reduce disease episodes is also affected by the reservoir of infection in the human liver stage (hypnozoites), which occurs even in the absence of the bite of infectious vectors [22]. Moreover, the infection rate of *P. vivax* was increased by 1.5% in September, one of the highest malaria peak months in the zone. This month is mainly associated with warm temperature and high precipitation in the study area [23], which favours the development of mosquitoes in the surrounding stagnant water permitting the high disease transmission.

Although significant difference was not observed from the model on *P. vivax* positivity rate, the interrupted time series analysis revealed slight reduction following the second round preventive measures in 2017. This might be attributed to the enhanced coverage of IRS and LLIN following the second round execution of these malaria control tools, which might prevented the new vivax infection from mosquito bite. Furthermore, recently the country has revised its malaria diagnosis and treatment guideline supposed to reduce malaria prevalence significantly. Accordingly, primaquine phosphate, the anti-relapse anti-malarial drug at a dose of 0.25 mg base per kg daily for 14 days combined with chloroquine (25 mg/kg for three consecutive days) has been recommended for radical cure of *P. vivax* mono-infection [24], which contributes to the reduced risk to vivax malaria.

The status of malaria among patients in different age groups of the study site between 2014 and 2019 showed the highest prevalence among adult patients aged > 15 years than children under five years. Similarly, in other retrospective studies conducted in different parts of the country showed similar pattern with high malaria infection observed among patients aged > 15 years [25–27]*.* The observed highest prevalence might be linked to the occupational activities of adults as they are usually engage in outdoor activities [28].

To sum up, in Ethiopia, malaria reaches its peak of infection from September to December following heavy rainy season, and April to May after the light rain. Finding of the study revealed that malaria peak season was irregular and varied from district to district and year-to-year. In 2014, malaria prevalence reached its peak in March to May, while in the years, 2016 to 2019, the highest infection was observed in months of June/July to September/October, rainy season in the area. In all years, in agreement with the general truth, the prevalence was relatively lower during dry season (from January to May), although the districts experience shorter rainy seasons and less intra-annual variability in temperature [29]. Rainfall influences malaria transmission as the rain water create favourable breeding habitats for the vector mosquito [30] and increase the rate of malaria transmission.

## Conclusion

The overall prevalence of malaria documented in the study area between 2014 and 2019 was significant. This finding substantiated the public view that malaria burden is still high in the study area in these years. This calls for further surveillance along careful planning, cautious designing of integrated interventional strategies mainly those targeted *P. vivax*, and ensuring close case surveillance by all the concerned public health authorities. Despite that, the observed reduction was promising and hoping that the nationally set malaria elimination goal by 2030 might be achieved, if this initiative become intensified and sustained by considering the peculiar nature of each *Plasmodium* species, and cases are carefully managed in all the districts. Unlike other regions of the country, the irregularity of malaria peak season in the study area demand special attention by the concerned bodies, as there might be a possibility of unexpected occurrence of outbreak in the future.

## Data Availability

The data used to support the findings of this study are all included/ available in the manuscript.

## Abbreviations

FMOH: Ethiopian Federal Ministry of Health
IRS: Indoor residual spraying
HH: Head house
ITN: Insecticide-treated mosquito net
LLINs: Long-lasting insecticidal nets
NMCEP: Ethiopia’s National Malaria Control and Elimination Programme
NMSP: National Malaria Strategic Plan
PMI: President’s Malaria Initiative
RDT: Rapid diagnostic tests
RR: Relative risk
WHO: World Health Organization
